# Common Neural System for Sentence and Picture Comprehension Across Languages: A Chinese–Japanese Bilingual Study

**DOI:** 10.3389/fnhum.2019.00380

**Published:** 2019-10-25

**Authors:** Zhengfei Hu, Huixiang Yang, Yuxiang Yang, Shuhei Nishida, Carol Madden-Lombardi, Jocelyne Ventre-Dominey, Peter Ford Dominey, Kenji Ogawa

**Affiliations:** ^1^Department of Psychology, Hokkaido University, Sapporo, Japan; ^2^INSERM - U1093 Cognition, Action, and Sensorimotor Plasticity, Dijon, France

**Keywords:** semantic processing, sentence comprehension, bilingualism, fMRI, MVPA

## Abstract

While common semantic representations for individual words across languages have been identified, a common meaning system at sentence-level has not been determined. In this study, fMRI was used to investigate whether an across-language sentence comprehension system exists. Chinese–Japanese bilingual participants (*n* = 32) were asked to determine whether two consecutive stimuli were related (coherent) or not (incoherent) to the same event. Stimuli were displayed with three different modalities (Chinese written sentences, Japanese written sentences, and pictures). The behavioral results showed no significant difference in accuracy and response times among the three modalities. Multi-voxel pattern analysis (MVPA) of fMRI data was used to classify the semantic relationship (coherent or incoherent) across the stimulus modalities. The classifier was first trained to determine coherency within Chinese sentences and then tested with Japanese sentences, and vice versa. A whole-brain searchlight analysis revealed significant above-chance classification accuracy across Chinese and Japanese sentences in the supramarginal gyrus (BA 40), extending into the angular gyrus (BA 39) as well as the opercular (BA 44) and triangular (BA 45) parts of the inferior frontal gyrus in the left hemisphere (cluster-level FWE corrected *p* < 0.05). Significant above-chance classification accuracy was also found across Japanese sentences and pictures in the supramarginal (BA 40) and angular gyrus (BA 39). These results indicate that a common meaning system for sentence processing across languages and modalities exists, and it involves the left inferior parietal gyrus.

## Introduction

Some of the languages in existence nowadays share similarities in phonological and/or orthographic properties, while others do not. However, all human beings are capable of acquiring another language besides their native language. Language is a symbolic representation of the knowledge of the world, the meaning which is also known as semantics in the domain of linguistics. It is possible to assume that the neurobiological infrastructure that is largely shared among humans is likely to be the neural system that underlines semantic processing (Binder et al., 2009; Hagoort, 2014).

The comprehension of semantics requires the automatic parallel processing of sound, word, and sentence patterns (Fromkin et al., 2014). Nevertheless, the semantic properties of words/sentences are readily distinguished from their structural properties (Binder et al., 2009). Also, the neural processing of different language structures is distinguishable (e.g., Tan et al., 2005; Grodzinsky and Friederici, 2006; Buchweitz et al., 2009), even as the brain regions overlap to some degree (e.g., Keller et al., 2001; Hickok and Poeppel, 2004).

Binder et al. (2009) reviewed neuroimaging studies (i.e., fMRI and PET studies) to identify brain regions that contribute to the semantic component of words and found that the left posterior parietal lobe, the lateral temporal cortex, and the inferior frontal gyrus demonstrated a high likelihood of activation across studies. Recent fMRI studies applied multi-voxel pattern analysis (MVPA) to investigate neural representations associated with semantics by analyzing patterns of neural activation (Mitchell et al., 2008; Shinkareva et al., 2011). This approach also enabled a comparison through which to investigate the common neural representations across people and languages (Zinszer et al., 2016; Yang et al., 2017a), especially in bilingual semantic processing. For example, the common neural representation of equivalent Portuguese and English nouns was found to be situated in the left post-central and supramarginal gyri (SMG), the left inferior and superior parietal lobes (I/SPL), the left inferior frontal gyrus (IFG), and the posterior superior temporal lobe (Buchweitz et al., 2012). Meanwhile Correia et al. (2014) argued that the shared representation across Dutch and English was located in the left anterior temporal lobe (ATL), the left angular gyrus (AG), the posterior bank of the left postcentral gyrus, the right posterior superior temporal sulcus/gyrus (STS/STG), the right anterior insula, the medial part of right ATL, and the bilateral occipital cortices. Van de Putte et al. (2017) investigated the common neural representation across French and Dutch and proved that the shared semantic representations are located in the bilateral occipito-temporal cortex and in the inferior and the middle temporal gyrus (ITG/MTG). Overall, the previous neuroimaging studies investigating the common neural representation of the semantic processing of words have yielded a consistent result, regardless of whether the participants were asked to read (Buchweitz et al., 2012), listen (Correia et al., 2014), or speak (Van de Putte et al., 2017) the words. Those studies suggested that a common neural representation might comprise a number of brain regions, including the left inferior parietal lobe (AG and portions of SMG) and the superior/middle temporal lobe. However, inconsistent results still existed, and this inconsistency might be resolved via using more natural processing, i.e., sentence processing.

In real life we communicate in written or spoken sentences formed of words that are arranged according to complicated syntactic rules (Ingram, 2007). Accordingly, the meanings conveyed by sentences transcend the individual words. However, the neural system underlying the semantic processing of sentences is still controversial. Price (2010) reviewed studies that have investigated the brain regions involved in the semantic processing of spoken sentences and argued that the neural system at the sentence level was situated in the anterior and posterior parts of the left middle temporal gyrus, the bilateral anterior temporal poles, the left AG, and the posterior cingulate/precuneus. These areas were associated with the semantic processing of words as reported by Binder et al. (2009). Interestingly, Jouen et al. (2015) identified a common neural system from processing whole sentences and images that describe human events that also includes the left AG. To our knowledge, however, only one MVPA study has argued the existence of commonalities in the neural system of bilinguals in the semantic processing of sentences across languages: Yang et al. (2017b) mapped the semantic properties of English words and their neural representations and subsequently developed a predictive model containing the neural system of sentences that were composed from these words. Although they demonstrated the above chance accuracy of predicting the activation pattern of Portuguese sentences from equivalent English sentences, the Portuguese–English bilinguals were presented with only their native Portuguese sentences. The direct prediction of simultaneous semantic processing between the two languages known by the bilinguals was not conducted. Also, as Yang et al. (2017b) have suggested, all the across-language neural decoding and prediction studies used stimuli that only encompassed a small semantic space. In other words, they used a limited number of concrete nouns to represent dwellings, tools, animals, or other objects. It is hence unknown whether the neural system of the vast semantic spaces across languages can be similarly predicted.

In order to achieve the understanding of the semantics of sentences precisely, the syntactic processing is necessary for dealing with fitnesses of different arguments of words and phrases (Bookheimer, 2002; Humphries et al., 2007). This syntactic processing is considered to be subserved by the pars opercularis (BA 44), a subpart of the left inferior frontal gyrus (IFG) (Newman et al., 2010; Friederici, 2011; Makuuchi and Friederici, 2013). Further, during the sentence comprehension, the syntactic information needs to be integrated with the semantic information (Marslen-Wilson and Tyler, 1980) that is subserved by the pars trianularis (BA 45) which is another subpart of the left IFG (Friederici, 2011). Thus, the integration processing of the syntactic and semantic information is assumed to be supported in the left IFG including BA 44 and BA 45 (Hagoort, 2005, 2014). However, some findings suggested that the region which supports the processing of the syntactic and semantic integration is located in the posterior temporal cortex (Friederici, 2011, 2012). Despite these controversies, we hypothesized that the common neural system of the semantic processing of sentences might comprise regions associated with the syntactic processing, which are located in the left IFG.

This study aimed to investigate the common neural system of the semantic processing of sentences across languages. Bilingual participants were asked to read both the Chinese and the equivalent Japanese sentences and to understand them. The cross-language classification was implemented. This application comprised training the support vector machine (SVM) classifier (Cortes and Vapnik, 1995) with sentences in one language and testing it with sentences in the other language and vice versa. Our analysis involved the training and testing of the SVM with sentences in one language. In addition, participants were presented with pictures that depicted the same kinds of human events as in the sentences. The participants thus performed the semantic processing of three different modalities: Chinese written sentences, Japanese written sentences, and pictures.

## Materials and Methods

### Participants

Thirty-two right-handed speakers of Chinese as their first language participated in the study. Behavioral results showed that the accuracy of three participants on either task condition was lower than the chance level (50%). Thus, these three participants were removed from both the behavioral and the fMRI analyses. The remaining 29 participants (6 males; mean age = 27.93, *SD* = 3.65) with normal or corrected to normal vision reported that they did not suffer from any neurological or psychiatric disorder. Each participant signed the informed consent format approved by the Ethical Committee of the Graduate School of Letters at Hokkaido University.

All the participants were late sequential bilinguals who began learning Japanese at the average age of 18.03 (*SD* = 3.26). They had been learning Japanese for 9.71 years (*SD* = 4.23) on average, and had been living in Japan for an average of 4.85 years (*SD* = 2.59). Except for one participant who had studied and lived in Japan for more than 10 years, all the other participants had passed the highest level of Japanese Language Proficiency Test. Twenty-two of the participants were enrolled in a graduate-level course, and one was registered in an undergraduate program at Hokkaido University. Seven of the participants were employed in occupations: of these, four were master’s degree holders, two had earned doctoral degree, and one passed the requirements for the bachelor’s degree.

The participants filled out a language self-rating questionnaire to help researchers ascertain their Japanese language proficiency. The questionnaire asked participants to award self-rating points on a scale of 1.0 (poor) to 7.0 (excellent). The questionnaire contained five questions each on listening, speaking, reading, and writing skills which were collected from the JLPT Can-do Self-Evaluation List created by the Japan Foundation and the Japan Educational Exchanges and Services^1^. Despite being late bilinguals, the participants rated themselves as being highly proficient in Japanese (*M* = 6.24, *SD* = 0.58).

### Stimuli

**FIGURE 1:**
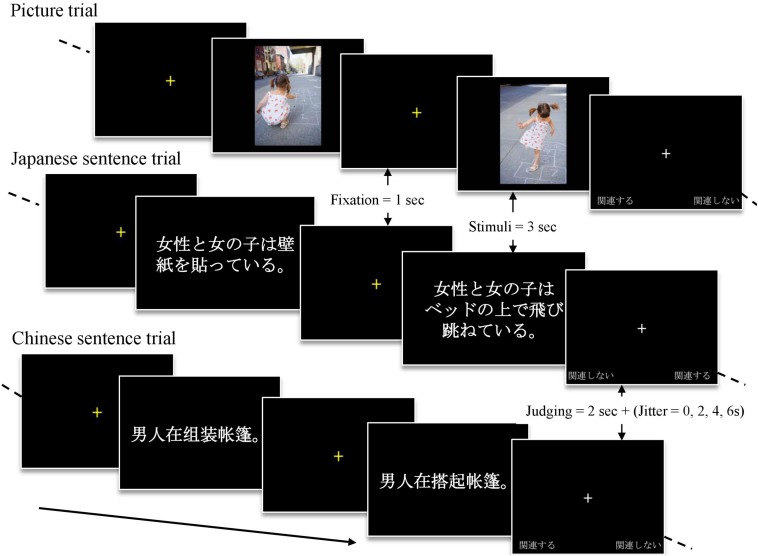
Experimental paradigm.

The stimuli comprised 48 pairs of pictures, and Chinese and Japanese written sentences totaling 144 pairs. The pictures (adapted from Jouen et al., 2015) depicted events representing one or two persons (no negative emotional valence) performing a common daily activity (e.g., playing the piano, cooking, reading a book to a child, etc.) and were collected from the Getty photo database^2^. A pair of pictures either symbolized a sequence of coherent events (for example, the first picture showed a girl throwing a piece of rock and the second picture portrayed the girl playing hopscotch as shown in Figure 1) or incoherent events (e.g., the first displayed a girl and a woman mounting wallpaper, and the second portrayed the same two characters jumping up and down on a bed), and the paired pictures represented only either coherent or incoherent events. Both the Chinese and Japanese sentences were generated on the basis of the pictures: the sentences described the activities being performed by the people in the pictures. The Chinese sentences were first generated and subsequently translated into equivalent Japanese sentences. The validity of the Japanese translation was confirmed by consulting with a native Japanese speaking expert. As a result, each event pair conveying the same meanings was represented by three different modalities: the picture, the Chinese sentence, and the Japanese sentence.

Three stimuli sets (A, B, and C) were generated, each comprising 16 picture pairs, 16 Chinese sentence pairs, and 16 Japanese sentence pairs. To avoid the participants seeing a picture pair and receiving its corresponding Chinese or Japanese sentence pair in a single session, stimuli pairs were crossed. For example, the 16 picture pairs were classified into set A, and their corresponding 16 Chinese and Japanese sentence pairs were then respectively classified into sets B and C.

The stimuli were balanced for coherency (coherent or incoherent) and the number of individuals performing the activity (one or two), and both were counterbalanced across the three stimuli sets. The Chinese sentences obeyed the subject–verb–object order and the Japanese sentences obeyed the subject–object–verb order according to the grammar rules of the respective languages. The Chinese sentences had a mean length of 10.08 words (*SD* = 2.62) and the Japanese sentences had an average of 16.33 words (*SD* = 3.38).

### Stimuli Evaluation

To assess the degree to which the sentences matched with the pictures, 20 pilot participants (10 Chinese natives and 10 Japanese natives) who did not participate in the fMRI experiment were recruited. These participants were presented with sentences in their respective native language and were asked to rate how appropriately the sentences were able to describe the activities being performed by the persons in the pictures on a scale of 1.0 (very poorly) to 7.0 (very well). The mean rating score was 6.51 (*SD* = 0.40) for the Chinese sentences and 6.69 (*SD* = 0.30) for the Japanese sentences. Both the Chinese and the Japanese sentences were thus rated as being excellently able to describe the activities in the pictures. In addition, the variety of activities in the pictures ensured that the Japanese used in the sentences would incorporate a vast semantic space. Insufficient knowledge of the used Japanese sentences would probably affect the semantic processing; therefore, the frequency of the use of the words of the Japanese sentences was evaluated. All the Japanese sentences were segmented into words (totally 179 words), and the incidence of the use of the particular words was investigated using the long-unit-word and the short-unit-word aspect of The Balanced Corpus of Contemporary Written Japanese (BCCWJ) (National Institute for Japanese Language and Linguistics, 2018). Except for the newly coined word “selfie,” the frequencies of the words used for the sentences ranked between extremely low and high levels of usage, extending from 0.16 to 48383.91 per million words.

### Procedures

Before the fMRI scanning session, participants completed the vocabulary checking list that was generated according to word frequency. The list contained 30 words collected in the order of decreasing word frequency. The participants were asked to remember the meanings of the words that they did not know to avoid interference with sentence comprehension caused by not understanding the words that were used.

During the scanning, participants underwent three sessions (i.e., stimuli sets A, B, and C), and the order of the sessions was counterbalanced across participants. Each session included 48 trials, and all the trials were randomly presented. During a trial, a yellow fixation cross appeared on a black background for 1 s, then the first stimulus of the event pair appeared on a black background for 3 s, followed by another yellow fixation cross for 1 s before the second stimulus of the event pair was presented for 3 s. At the end of this sequence, an evaluation screen with a white fixation cross containing options on a black background was presented for 2 s plus jitter time (0, 2, 4, or 6 s) before the next trial (Figure 1). In the trials in which event pairs were presented either in Chinese or Japanese, participants were instructed to silently and consistently read the sentences until they disappeared. In the trials in which the event pairs were presented in pictures, participants were instructed to continue thinking about the activities that the persons in the pictures were performing. In each trial, participants were also asked to judge whether or not the event pair was coherent by pressing a response button with their right index or middle finger as soon as they could after the evaluation screen appeared. The evaluation screen did not disappear after they had pressed this button. The judging options were arranged in accordance with the participants’ fingers: the alternative presented in the left bottom corner corresponded to the index finger and the selection appearing in the right bottom corner corresponded to the middle finger. The locations of the options were counterbalanced across the stimuli.

### fMRI Acquisition

Functional and structural image acquisition was performed on a Siemens Prisma 3.0 T scanner using a 64-channel head coil at the Research and Education Center for Brain Science of Hokkaido University. The whole brain functional images were collected using a T2^∗^-weighted gradient EPI sequence (*TR* = 2000 ms, *TE* = 30 ms, voxel size = 2 × 2 × 3.5 mm, and a 90° flip angle). A session consisted of 318 volumes. The high-resolution structural images covering the whole brain were acquired after the functional image acquisition using a T1 MPRAGE sequence (*TR* = 2300 ms, *TE* = 2.41 ms, *TI* = 900 ms, *FOV* = 256 × 256 mm, and an 8° flip angle).

### fMRI Data Preprocessing

fMRI data processing and analysis were performed with SPM 12 (Wellcome Department of Cognitive Neurology, London, United Kingdom) in the Matlab environment. The first three scans of all the sessions were removed from the analysis to minimize T1 artifacts. The functional images were corrected for slice timing and were spatially realigned to normalize to the Montreal Neurological Institute (MNI) space without changing the voxel size. Spatial smoothing was applied using a Gaussian kernel of 6 × 6 × 6 mm full width at half-maximum for univariate analysis. To prevent the possibility of less predictive individual voxels, spatially normalized but unsmoothed images were used to perform MVPA.

### fMRI Data Analysis: Univariate Analysis

To reveal neural regions generally involved in the semantic processing of Chinese sentences, Japanese sentences, and pictures, the general linear model (GLM) (Friston et al., 1995) was used to obtain contrasts between each modality and the baseline. The three different modalities were modeled as three separate regressors and were convolved with a canonical hemodynamic response function for each participant. The group analysis was then processed through a second-level random effects model by using a one-sample *t*-test in a group analysis of all the participants. The activated regions were extracted with a cluster-level (*k* ≥ 15) threshold of *p* < 0.05 corrected for family-wise error (FWE).

### fMRI Data Analysis: Searchlight MVPA

A searchlight method (Kriegeskorte et al., 2006) with a linear SVM classifier, as implemented by LIBSVM (Chang and Lin, 2011), was performed to investigate the common semantic neural system across languages and modalities in the processing of sentence comprehension. A spherical searchlight with a radius of 9 mm was used to reveal multiple patterns that carried featured neural representations of the sentence semantics.

The classifier for the classification analysis was trained to discriminate between the neural patterns associated with the coherent and incoherent events. Two types of classification analysis were performed. First, a within-language/modality classification was accomplished with (1) only Chinese sentence trials, (2) only Japanese sentence trials, and (3) only picture trials. In all the within-language/modality classifications, a leave-one-out procedure was used: the classifier was trained on the data from any two of the three sessions and was tested on the data from the one session left. The classification was repeated thrice by interchanging the training and testing data. The accuracies were averaged across the three iterations.

The second cross-classification was accomplished across languages/modalities classification with (4) Chinese vs. Japanese sentences, (5) Chinese sentence vs. picture, and (6) Japanese sentence vs. picture. The classifier was trained with data from one language/modality condition belonging to all the three sessions. It was tested on the respective data obtained from the remaining language/modality condition belonging to all the three sessions. Each classification was repeated twice in a manner ensuring that all of the specific language/modality data were used once for the test. The resulting accuracies were averaged across classification directions.

To construct accuracy group maps for across-language/modality, the accuracies were averaged across all participants and contrasted with the average accuracy of the coherency (accuracy at chance = 50%) using a one-sample *t*-test to reveal the cluster level (*k* ≥ 10) significant classification of sentence semantics across languages/modalities (*p* < 0.05, FWE-corrected). Group maps were also produced for the within-language/modality (*p* < 0.05, FWE-corrected, *k* ≥ 10).

To reveal a more robust result for the searchlight MVPA analysis, statistical maps were corrected using threshold free cluster enhancement (TFCE; Smith and Nichols, 2009) as implemented in a free MatlabTFCE package^3^ which combined a maximal permuted statistic correction technique (Nichols and Holmes, 2001). Ten thousand permutations and a one-tailed corrected cluster threshold of *p* = 0.05 were used (Wurm and Caramazza, 2019).

### fMRI Data Analysis: Region of Interest (ROI) Analysis

An ROI analysis was further performed to specifically investigate the effects of regions that are commonly activated during sentence processing. ROIs were selected based on Jouen et al. (2015) study, which investigated the common neural representations for the semantic processing of sentences in monolinguals by performing conjunction analysis between written sentences and pictures. Regions such as BA 22 (superior temporal gyrus), BA 39 (AG, inferior parietal lobe), and BA 45 (triangular part of inferior frontal gyrus) were selected on the basis of their reporting of these areas being involved in the semantic processing of sentences. Two further regions were selected: BA 40 (supramarginal gyrus), which is adjacent to BA 39, and BA 44 (the opercular part of the inferior frontal gyrus), which is adjacent to BA 45. These regions are considered to be part of the classical language region and are known as Wernicke’s area and Broca’s area, respectively (Catani et al., 2005; Hagoort, 2017). To assure that the sentences were processed as semantic stimuli rather than visual stimuli, BA 17 (primary visual cortex) was also selected, and it was proved to be involved in visual information processing as the control region.

## Results

### Behavioral Results

**FIGURE 2:**
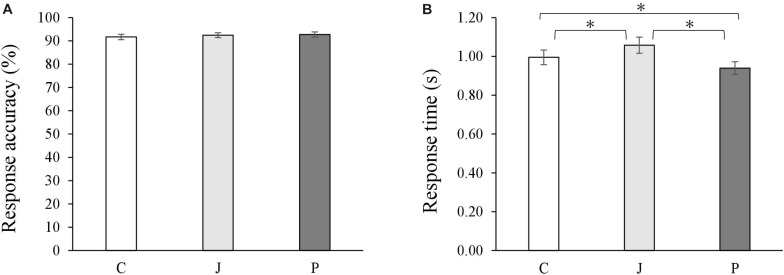
Average accuracies **(A)** and response time **(B)** on judging coherence of stimuli of all conditions. Bars represent means with standard errors. C represents the Chinese sentence condition, J represents the Japanese sentence condition, and P represents the picture condition (^∗^*p* < 0.05).

During the fMRI scanning, the participants performed a coherence judging task. In all the conditions involving the Chinese sentence, the Japanese sentence and the picture, they evaluated the coherence with a high accuracy (Chinese sentence: *M* = 92%, *SEM* = 0.01; Japanese sentence: *M* = 92%, *SEM* = 0.01; picture: *M* = 93%, *SEM* = 0.01; Figure 2A). No significant differences were found on the accuracy [*F*(2,56) = 0.56, *p* = 0.58, $\eta_{p}^{2}$ = 0.02].

A significant difference was found pertaining to the condition on the response time [*F*(2,56) = 26.95, *p* < 0.001, $\eta_{p}^{2}$ = 0.49]. Participants responded faster to the picture (*M* = 0.94s, *SEM* = 0.03) than to the Chinese sentence (*M* = 1.00s, *SEM* = 0.04) and to the Japanese sentence (*M* = 1.06s, *SEM* = 0.04; Figure 2B). In a supplementary analysis, differences in the accuracy and the response time of the coherence judgment for all the conditions were analyzed (see Supplementary  Material).

### Univariate Analysis

We accomplished a voxel-based analysis of the whole brain activation to reveal activated neural regions for semantic processing of the Chinese sentence, the Japanese sentence, and the picture (*p* < 0.05, FWE-corrected, ke ≥ 15).

**FIGURE 3:**
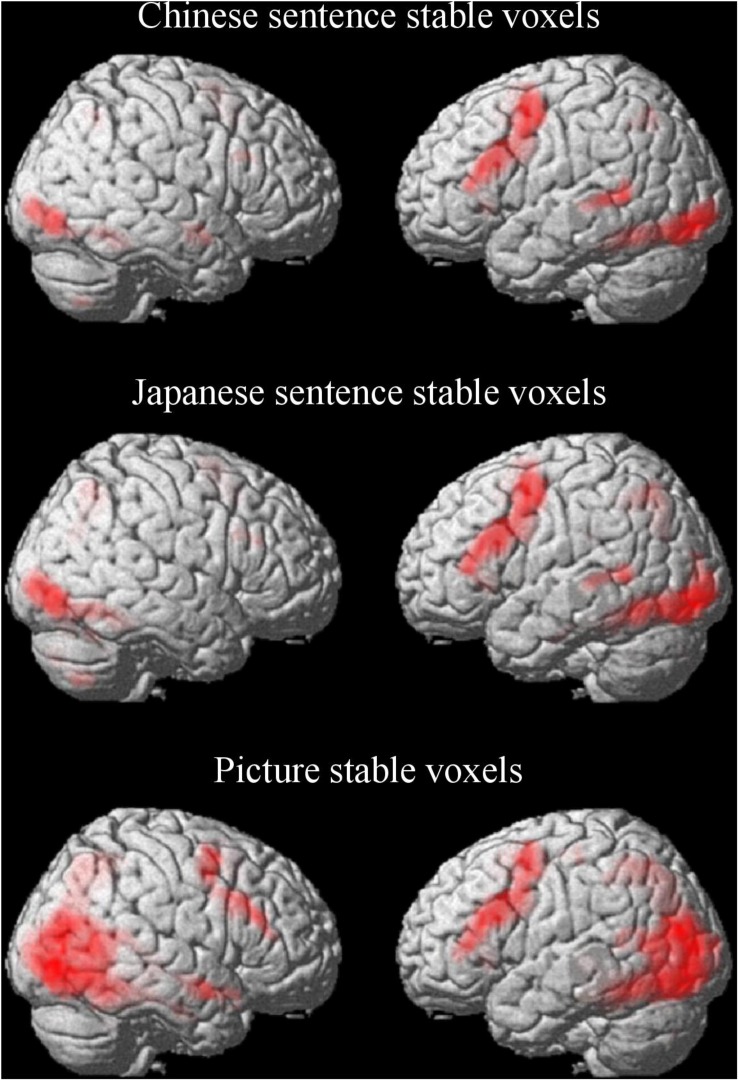
Stable voxels for the Chinese sentence, Japanese sentence and picture in semantic processing. Stable voxel clusters significant at *p* < 0.05, FWE-corrected, extent threshold = 15 voxels.

For the semantic processing of the Chinese sentence, a large predominantly left-hemisphere network was activated (Figure 3 and Table 1A). These regions included clusters spreading from the precentral gyrus (BA 6) and the supplementary motor area (SMA; BA 6) to the opercular part of the inferior frontal gyrus (Oper-IFG; BA 44); from the lateral inferior occipital gyrus (IOG; BA 18) to the fusiform gyrus (BA 37); and from the middle temporal gyrus (MTG; BA 21/22) to the superior parietal gyrus (SPG; BA 7) in the left hemisphere. The clusters that spread from the fusiform gyrus (BA 37) to the lingual gyrus (BA 18) and calcarine cortex (BA 17), and areas which included the MTG (BA 20) and the triangular part of the inferior frontal gyrus (Tri-IFG) in the right hemispheres were also found.

**TABLE 1:** Activation for Chinese sentence **(A)**, Japanese sentence **(B)** and picture **(C)** semantic processing.

	**Voxels**	**BA**	**T**	**MNI**		
				**x**	**y**	**z**
**(A) Chinese sentence**						
L precentral gyrus	263	6	14.74	–42	2	47
L opercularis inferior frontal gyrus			12.04	–45	17	23
L opercularis inferior frontal gyrus		44	11.96	–45	5	29
L inferior occipital gyrus	565	18	14.37	–15	–94	–7
L fusiform gyrus		37	14.01	–45	–55	–16
L inferior occipital gyrus		18	13.21	–24	–94	–7
L supplementary motor area	204	6	12.36	–3	2	62
L supplementary motor area			11.81	–3	8	53
R middle cingulum gyrus			6.19	9	20	44
L middle temporal gyrus	132	21	11.37	–54	–49	8
L middle temporal gyrus		22	10.35	–57	–37	5
L insula	32	47	9.98	–30	23	2
L superior parietal gyrus	60	7	8.64	–27	–64	50
L cerebellum	21		7.79	–3	–52	–37
R fusiform gyrus	347	37	12.05	39	–49	–19
R lingual gyrus		18	11.94	18	–85	–7
R calcarine cortex		17	11.20	15	–91	–1
R cerebellum	18		10.58	30	–67	–52
R middle temporal gyrus	40	20	7.70	51	–10	–13
R triangularis inferior frontal gyrus	26		7.34	39	23	26
**(B) Japanese sentence**						
L inferior occipital gyrus	832	18	16.93	–15	–94	–7
L inferior occipital gyrus		18	14.48	–24	–94	–7
L fusiform gyrus		37	14.04	–42	–58	–13
L precentral gyrus	680	6	15.42	–42	2	47
L opercularis inferior frontal gyrus			12.64	–51	17	23
L precentral gyrus		44	12.39	–45	5	32
L supplementary motor area	22	6	12.16	–3	2	62
L supplementary motor area		32	11.10	–3	11	50
L insula	47	47	11.22	–30	23	2
L superior parietal gyrus	200	7	10.68	–27	–64	50
L middle occipital gyrus		19	7.87	–27	–70	29
L middle temporal gyrus	103	21	10.22	–54	–49	8
L middle temporal gyrus		22	9.09	–54	–37	5
L thalamus	15		8.34	–9	–13	5
L putamen	31		8.16	–21	2	5
R inferior occipital gyrus	675	19	13.70	30	–82	–13
R lingual gyrus		18	13.24	18	–82	–10
R calcarine cortex		17	12.52	15	–91	–1
R angular gyrus	99	7	9.90	30	–61	50
R middle occipital gyrus		19	8.50	33	–70	26
R cerebellum	27	37	9.12	30	–61	–28
R triangularis inferior frontal gyrus	30		7.02	42	26	26
**(C) Picture**						
L supplementary motor area	197	32	12.93	–3	14	50
L triangularis inferior frontal gyrus	675		12.35	–45	26	23
L opercularis inferior frontal gyrus		44	11.38	–45	5	29
L precentral gyrus		6	10.53	–42	5	44
L insula	36	47	9.93	–30	23	2
L postcentral gyrus	19		7.70	–39	–25	59
L cerebellum	16		7.54	–9	–73	–25
L cerebellum	16		7.49	–9	–76	–40
R hippocampus	5103		20.97	24	–28	–4
L fusiform gyrus		37	18.56	–39	–58	–16
L fusiform gyrus		37	18.21	–27	–49	–10
R precentral gyrus	342	6	11.68	39	2	47
R triangularis inferior frontal gyrus			10.29	48	29	23
R middle frontal gyrus		6	9.73	36	2	56
R middle temporal gyrus	127	21	9.78	60	2	–16
R superior temporal gyrus		22	9.60	54	–7	–13
R superior temporal pole		38	9.10	48	14	–19
R amygdala	38	34	9.16	30	–1	–19
R hippocampus		35	9.00	21	–7	–19

*Clusters of voxels significant at *p* < 0.05, FWE-corrected, extend threshold = 15 voxels. Region labels apply to the entire extent of the cluster with peak maxima designated by first locale cited.*

Similarly, for the semantic processing of the Japanese sentence, the left-hemisphere regions were predominantly activated and partially overlapping with the semantic processing of the Chinese sentence (Figure 3 and Table 1B). Clusters in the left hemisphere extended from the IOG (BA 18) to the fusiform gyrus (BA 37), from the precentral gyrus (BA 6) and the SMA (BA 6) to the Oper-IFG (BA 44), from the SPG (BA 7) to the MOG (BA 19), and the MTG (BA 21/22). In the right hemisphere, the clusters encompassed were the IOG (BA 19) to the lingual gyrus (BA 18) and the calcarine cortex (BA 17), as well as the AG (BA 7) to the MOG (BA 19), and the Tri-IFG.

In opposition to the Chinese and Japanese sentence processing, the semantic processing of the picture activated regions more bilaterally (Figure 3 and Table 1C). Except for the cluster spreading from the superior temporal pole (BA 38) via the superior temporal gyrus (STG; BA 22) to the MTG (BA 21) in the right hemisphere, the regions that were activated both in the left and right hemispheres were symmetrical to some degree. The first two regions were the cluster extended from the internal SMA (BA 32) and the precentral gyrus (BA 6) to the Tri-IFG (BA 44) in the left hemisphere, and the cluster extended from the precentral gyrus (BA 6) and the middle frontal gyrus (MFG; BA 6) to the Tri-IFG in the right hemisphere. The other two regions were the broad areas located in the occipital lobe spreading from the left fusiform gyrus to the right hippocampus as the peak locations.

Furthermore, we observed the greater activity in the left inferior parietal gyrus (BA 40), the right supramarginal gyrus, and middle occipital gyrus for coherent compared with incoherent semantic processing (details are provided in the Supplementary  Material).

### Searchlight MVPA Analysis

#### Within-Language/Modality Classification

**FIGURE 4:**
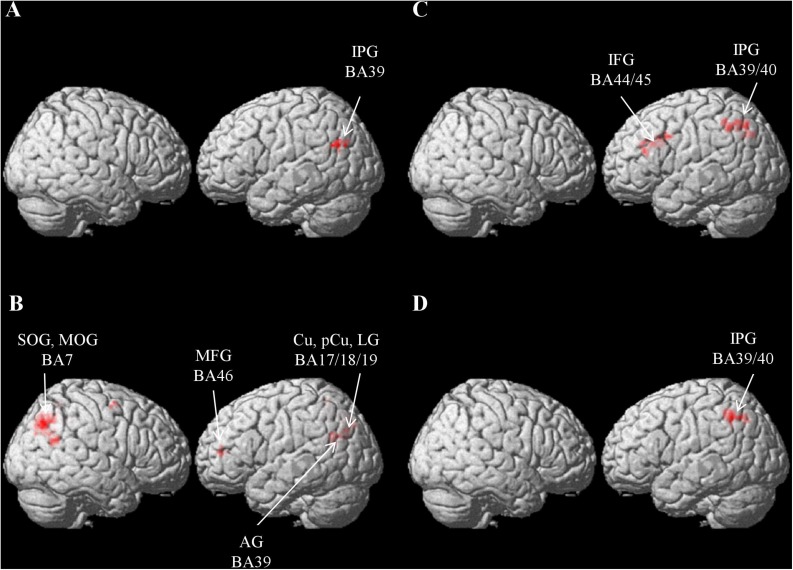
Results of the searchlight analysis, showing significant classification accuracies above chance level (50%) from averaged whole-brain maps from all the participants at a cluster level FWE corrected at *p* < 0.05. **(A)** Results for within-Chinese classification. **(B)** Results for within-picture classification. **(C)** Results for across-language classification. **(D)** Results for across-modality classification.

Figure 4A and Table 2A exhibit the areas in which the significant classification accuracies were found for the Chinese sentence. These regions were located in the left AG (BA 39) and extended to the MOG (BA 19). No significant classification accuracies were found within the Japanese sentence.

**TABLE 2:** Brain areas showed significant across-language **(A)**, across-modality **(B)**, within-Chinese **(C)** and within-picture **(D)** classification accuracies.

	**Voxels**	**BA**	**T**	**MNI**		
				**x**	**y**	**z**
**(A) Across languages**						
L inferior parietal gyrus	47	40	5.93	–42	–46	41
L inferior parietal gyrus		40	4.93	–48	–46	50
L angular gyrus		7	4.81	–36	–67	44
L precentral gyrus	36	44	5.07	–48	11	38
L triangularis inferior frontal gyrus		45	4.83	–45	32	20
L triangularis inferior frontal gyrus			4.61	–42	26	29
**(B) Across Japanese and picture**						
L inferior parietal gyrus	48	40	6.30	–45	–55	47
L angular gyrus		39	5.24	–39	–61	47
L inferior parietal gyrus		40	4.78	–42	–46	50
**(C) Within Chinese**						
L angular gyrus	22	39	4.43	–45	–61	29
L angular gyrus		39	4.41	–42	–58	26
L middle occipital gyrus		39	4.36	–42	–70	26
L angular gyrus		39	4.16	–48	–64	26
L angular gyrus		39	3.85	–48	–58	26
L middle temporal gyrus		39	3.82	–45	–64	20
L middle occipital gyrus		19	3.77	–36	–70	32
**(D) Within picture**						
L cuneus	16	19	6.41	–12	–79	38
L cuneus		18	3.65	–15	–73	32
L precuneus	14	5	5.19	–3	–52	62
L precuneus			4.74	–3	–58	47
L angular gyrus	13	39	4.60	–42	–55	23
L angular gyrus		39	4.50	–48	–61	29
			3.86	–39	–61	17
L lingual gyrus	11	17	4.60	–3	–64	8
L lingual gyrus		17	4.20	–9	–76	2
L calcarine cortex		17	3.69	–9	–70	8
L middle occipital gyrus	14	19	4.38	–33	–67	29
L middle frontal gyrus	11	46	4.12	–36	47	14
R middle occipital gyrus	200	19	7.84	39	–70	35
R superior occipital gyrus		7	7.64	30	–70	41
R precuneus		7	7.01	9	–73	41

*Clusters of voxels significant at *p* < 0.05, FWE-corrected. Region labels apply to the entire extent of the cluster with peak maxima designated by first locale cited.*

Areas involved in the classification of the pictures were more bilateral (Figure 4B and Table 2B) and included the left parieto-occipital regions spreading from the AG (BA 39) to the cuneus (BA 18/19), precuneus (BA 5), the lingual gyrus (BA 17/19), and the left MFG (BA46). The right MOG extending to the superior occipital gyrus (SOG; BA 7) and the precuneus (BA 7) were also noted.

#### Across-Language/Modality Classification

Significant across-language (i.e., Chinese sentence vs. Japanese sentence) classification accuracies were found in the left inferior parietal gyrus (IPG), which extends from the supramarginal gyrus (SMG; BA 40) to the AG (BA 39/7), and in the left precentral gyrus extending to the Oper and Tri-IFG (BA 44/45; Figure 4C and Table 2C).

Significant across-modality (i.e., Japanese sentence vs. picture) classification accuracy involved the left IPG extending from the SMG (BA 40) to the AG (BA 39/7) (Figure 4D and Table 2D). No significant classification accuracy was found between the Chinese sentence and the picture.

Results of using the TFCE also showed significant above chance classification accuracies for within- and across-language/modality classification. In the within-picture classification, significant classification accuracies were found in bilateral parieto-occipital regions (Supplementary Figure 4A). In the across-language classification, significant classification accuracies were found in the left IPG and the left IFG (Supplementary Figure 4B), though the regions significantly activated were smaller than those obtained using the searchlight MVPA. In contrast, significant classification accuracies were not observed for the within-Chinese sentences and the Japanese vs. picture classifications.

### ROI Analysis

The mean classification accuracies in each ROI were contrasted with the chance level of accuracy (50%) using a one-sample *t*-test to accomplish the ROI analysis. For the within-Chinese sentence, the left BA 22 and BA 45 showed significant classification accuracies. For the within-Japanese sentence, the left BA 40, right BA 22, and BA 39 revealed significant classification accuracies. For the within-picture, the significant classification accuracies were shown in the bilateral BA 22, BA 39, and BA 40, the right BA 39, and the left BA 17, which was involved in primary visual information processing.

**FIGURE 5:**
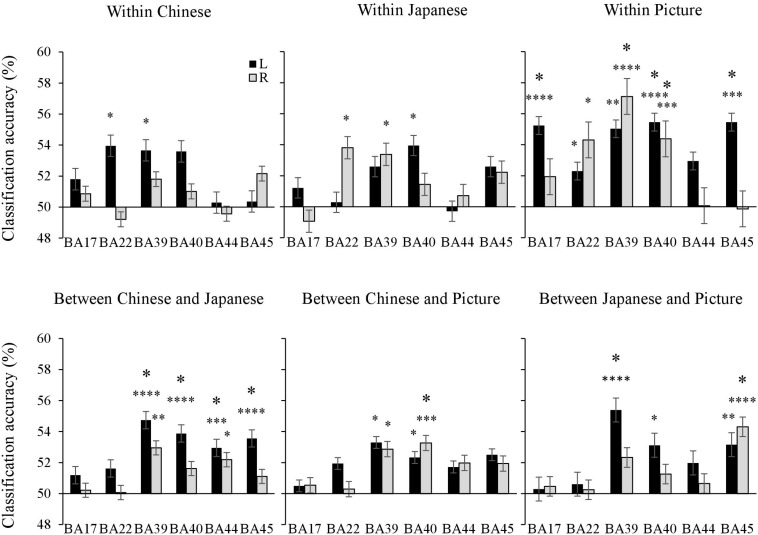
The mean classification accuracies in each ROI were contrasted with the chance level of accuracy (50%) using a one-sample *t*-test. Bars represent standard errors. The small and large asterisks indicate statistical significance of *p* values uncorrected and corrected for multiple comparisons using Holm-Bonferroni procedure. ^∗^*p* < 0.05, ^∗∗^*p* < 0.01, ^∗∗∗^*p* < 0.001, ^∗⁣∗⁣∗∗^*p* < 0.0001.

For the across-language classification, significant classification accuracies were shown in the bilateral BA 44, the left BA 39, BA 40, and BA 45. Across modalities (i.e., Japanese sentence vs. picture), significant accuracies were shown in the left BA 39 and BA 40, and in the right BA 45. In contrast to the searchlight analysis, the ROI analysis revealed significant cross-modality classification accuracies for the Chinese sentence vs. the picture in the bilateral BA 39 and BA 40 (Figure 5).

## Discussion

The present study used MVPA to investigate the common neural system of the semantic processing during sentence comprehension across languages in bilinguals. The significant classification accuracies indicate the existence of a common neural semantic representation in the higher language processing level. More specifically, the common neural representation was found to be situated in the left inferior parietal gyrus extending from the angular gyrus to the supramarginal gyrus, and the opercular and triangular part of the left inferior frontal gyrus. The results of this study also suggest that the left inferior parietal gyrus, in particular, the left angular gyrus and supramarginal gyrus, is pivotal to the processing of semantics regardless of the modality.

### Across Languages

The univariate analysis showed similar brain activation associated with the processing of the same sentence semantics for both Chinese and Japanese. This suggests that a common neural representation may exist across languages but could not allow the identification of the exact regions for which the MVPA was conducted.

Despite the inconsistent results revealed by previous studies investigating the common neural representation of word semantic processing across languages using the MVPA approach (Buchweitz et al., 2012; Correia et al., 2014; Van de Putte et al., 2017), it is possible to assume that the left temporoparietal conjunction regions are highly involved in the semantic processing of languages. Indeed, these regions were active when subjects were exposed to sentences or pictures depicting human events (Jouen et al., 2015). The present study observed significant classification accuracies in the left IPG, i.e., the AG (BA 39) and the SMG (BA 40), across languages for the semantic processing of sentences. Accordingly, the role of the left AG and SMG in the semantic processing of languages is suggested regardless of the processing level (word or sentence).

The semantics of languages are conveyed by symbols such as characters and/or sounds, which can be combined into words and/or sentences. Though the processes are divergent in the initial stages, the visual and auditory information must be mapped onto meanings to understand a sentence (Humphries et al., 2007). Thus, the neural pathways underpinning the visual and auditory semantic processing converge in the left AG (Bemis and Pylkkänen, 2013) for comprehension. Further, a series of further semantic processes are executed in the left AG to understand exactly the meanings conveyed by the words, especially by the sentences (Humphries et al., 2007). The most crucial process is adjusting the input information by verifying the already-existing knowledge. This manipulation may be executed in the left AG through retrieving the knowledge of the objects and events where it stored (Binder et al., 2009; Binder and Desai, 2011; Noonan et al., 2013). Then, the word semantic judging (Bonner et al., 2013) and naming and reading of the word (Seghier et al., 2010) could be executed. Constructing the meanings of the individual words (Price et al., 2016), the sentence could be comprehended (Pallier et al., 2011). Our findings reconfirm the established importance of the left AG in semantic processing, and implicate that the left AG is a critical region for semantic processing of languages transcending different languages and the processing levels. Meanwhile, the semantics conveyed by the sentences presented in the study concerned events in which entities interacted in space and time. Therefore, it also suggests that the left AG is undoubtedly necessary in the processing of event-related semantics (Binder and Desai, 2011; Seghier, 2013; Jouen et al., 2015; Baldassano et al., 2017).

The SMG, another part of the left IPG that is anterior to the AG, was also observed in significant classification accuracy across languages. This region is traditionally considered to underlie phonological processing such as phonological recognition, phonological control, and production (Booth et al., 2004; Prabhakaran et al., 2006). The SMG probably contributes preferentially to phonological aspects during word recognition. On the other hand, it is also reported to be critical for semantic processing (Stoeckel et al., 2009), especially in reading, which requires the recognition of visual stimuli and their linking to meanings (Sliwinska et al., 2012) as accomplished in our study. Further, the posterior part of the SMG is suggested to be the area where lexical and sublexical cues are integrated (Oberhuber et al., 2016), the lexical phonological retrieval is controlled and from the orthography to phonology is mapped (Price, 2018), and the meanings processed in the AG are bound to recognize the word (Lee et al., 2007). In the semantic processing of the sentence where words were formed, it is possible to assume that the continuous linking of lexical cues and meanings is required. Overall, as our study has indicated, the cooperation of both the left AG and SMG in the semantic processing of the sentence is demanded.

Another significant classification accuracy across languages was observed in divergence with previous MVPA studies of the semantic processing of words in the left inferior frontal gyrus, i.e., Oper-IFG (BA 44) and Tri-IFG (BA 45) which constitute Broca’s area, a classical language processing area. Broca’s area might underlie not only the language production but also various other language functions (Geschwind, 1970; Kim et al., 1997), one of them being the executive control of language. Apart from the comparatively simple processing such as the semantic processing of a sentence, the participants of this study were required to maintain the meanings of the first stimulus of the stimuli pair while processing the second stimulus to complete the evaluation task. Further, processes such as lexical retrieval and matching with previously held knowledge were needed to be executed simultaneously to accomplish the exact comprehension of the meanings. All the execution of these above processes is considered to be mandated by Broca’s area (Devlin et al., 2003; Whitney et al., 2011; Noonan et al., 2013; Ralph et al., 2017).

Another role of Broca’s area is the syntactic processing of language (Caplan, 2006; Grodzinsky and Santi, 2008; Friederici, 2011); BA 44 is especially considered to be the core region of the syntactic processing (Friederici, 2011, 2012) that provides strong cues for determining meanings (Humphries et al., 2007). As hypothesized, this syntactic processing-associated region was observed in the study. This may explain the discrepancy from the previous MVPA studies and indicate that the syntactic processing is critical for the sentence comprehension. Toward the determination of meanings of sentences, the syntactic information decoded by analyzing the constructions of the words and phrases which form the sentences will be mapped with the semantic information (Bookheimer, 2002; Friederici, 2011). Because of the preferential contribution of BA 44 to the syntactic processing, BA 44 is necessary to interact with the region that subserves the semantic processing, which is considered to be BA 45 (Friederici, 2011) to determine meanings. Based on the results of the present study, it is possible to assume that syntactic and semantic integration occur in Broca’s area as Hagoort (2005, 2014) suggested. In this processing, the syntactic working memory is also required, which is considered to be one role of Broca’s area (Fiebach et al., 2005; Makuuchi and Friederici, 2013). In the higher level of the semantic processing of language performed in our study, besides the semantic processing, sentence comprehension also demands both the executive control and the syntactic processing. Therefore, the involvement of Broca’s area is presumable.

Broca’s area presented more superior extension to the ventral part of the precentral gyrus (BA 6) in the present study. As Hagoort (2005, 2014) suggested, it is more appropriate to refer to the BA 44, BA 45, BA 47, and BA 6 of the left inferior frontal cortex as Broca’s area, because adjacent areas such as BA 47 and BA 6 are also involved in language processing. This finding of the present study is greatly consistent with Hagoort’s assumption. Likewise, this finding might indicate the relevance of the motor-related system (i.e., BA 6) during comprehension of action-related sentences (e.g., Hauk et al., 2004; Hauk and Pulvermüller, 2011; Jouen et al., 2015), and the activation of acoustic representations during speech comprehension (Hickok and Poeppel, 2004, 2007; Cheung et al., 2016). Meanwhile, Broca’s area is connected via the superior longitudinal fasciculus with the left AG and SMG. Hence, it makes sense that the robust neural representation associated with the higher level of semantic processing is situated in the left inferior parietal gyrus (i.e., the AG and the SMG) and the left inferior frontal gyrus (BA 44 and 45) (Horwitz et al., 1998; Frey et al., 2008; Kelly et al., 2010).

### Across Modalities

A significant classification accuracy was observed in the left IPG (i.e., the AG and SMG) for semantic processing across modalities (i.e., Japanese sentence vs. picture and Chinese sentence vs. Japanese sentence). This result revealed the modality-independent common neural representation. The univariate analysis of the coherence judgment (see Supplementary Material) also showed the involvement of the left SMG for coherent semantic processing regardless of the modalities. These findings tend to support the idea proposed by Damasio (1989) and Meyer and Damasio (2009) that there are convergence zones where the features associated with different objects and events and/or information conveyed by different sensory systems are bound. The features and/or information were considered to be the processing of the meanings of the features and/or the information (Mahon and Caramazza, 2008). Despite the fact that the neural basis of the convergence zone is still controversial, the association of the temporoparietal regions overlapped to some degree (Jefferies, 2013; Simanova et al., 2014; Jouen et al., 2015; Wurm and Caramazza, 2019). Specifically, it is posited that the inferior parietal lobe, the ventral and lateral temporal lobes are involved in the higher-level convergence processing where the binding representation from multiple modalities encode an abstract or schematic concept (Binder and Desai, 2011; Simanova et al., 2014). Sentence comprehension requiring fluent conceptual combinations as in the present study demands the higher-level convergence processing of complex information. The information from the languages and modalities needs to be integrated with the stored knowledge in the convergence zone, which is the left IPG (Lau et al., 2008; Binder et al., 2009; Binder and Desai, 2011; Bonner et al., 2013; Seghier, 2013). Though the searchlight analysis evidenced the absence of significant classification accuracy between the Chinese sentence and the picture, the ROI analysis showed significant classification accuracy in the left BA 39 and BA 40. Hence, the results of the present investigation make it possible to indicate that the left inferior parietal gyrus (BA 39 and 40) is a modality-independent convergence zone for higher semantic processing.

## Conclusion

This study aimed to investigate whether an across-language sentence comprehension system exists using MVPA with Chinese–Japanese bilinguals, and whether such a system shares a common foundation for the broader comprehension of meaning in images. The results first suggest that the existence of the common neural system across languages in the semantic processing of sentences is located in the left inferior parietal gyrus (BA 39 and BA 40) and in the left inferior frontal gyrus (BA 44 and BA 45), which is also known as Broca’s area. Second, the findings elucidate the specific functioning of the left inferior parietal gyrus as a modality-independent convergence zone, particularly in higher semantic processing as required for understanding sentences and images.

## Data Availability Statement

The datasets generated for this study are available on request to the corresponding author.

## Ethics Statement

This study was carried out in accordance with the recommendations of the Hokkaido University Institutional Review Board with written informed consent from all subjects. All subjects provided written informed consent in accordance with the Declaration of Helsinki. The protocol was approved by the Hokkaido University Institutional Review Board.

## Author Contributions

ZH and KO conceived and designed the experiment. ZH performed the experiment, analyzed the data, and drafted the manuscript. HY and SN coordinated the data analysis. YY assisted in performing the experiment and collected the data. CM-L, JV-D, and PD contributed to conceptualization of the experiment and provided the material. PD reviewed the manuscript. KO supervised the experiment and the data analysis, reviewed and revised the manuscript.

## Conflict of Interest

The authors declare that the research was conducted in the absence of any commercial or financial relationships that could be construed as a potential conflict of interest.

## Footnotes

1 http://www.jlpt.jp/about/candolist.html

2 https://www.gettyimages.fr/

3 https://github.com/markallenthornton/MatlabTFCE
