# Supplementary material for: Common Neural System for Sentence and Picture Comprehension Across Languages: A Chinese–Japanese Bilingual Study
Source: Front Hum Neurosci. 2019 Oct 25;13:380. doi: 10.3389/fnhum.2019.00380 (PMC6823717; doi:10.3389/fnhum.2019.00380)
Supplement: Supplementary file 1 [file Table_1.docx]

Supplementary Material

# Behavioral results

To differentiate the effects of coherence (coherent or incoherent) on the event stimuli across all the conditions, we conducted a two-way analysis of variance. As reported, no significant main effect of all the conditions on accuracy was found (*F* (2, 56) = 0.63, *p* = 0.53, $\eta_{p}^{2}$ = 0.02), and there was no significant effect for the coherence (*F* (1, 28) = 0.21, *p* = 0.65, $\eta_{p}^{2}$ = 0.01). The interaction effect of all the conditions and the coherence was significant (*F* (2, 56) = 13.82, *p* < 0.001, $\eta_{p}^{2}$ = 0.33). In the Chinese sentence coherence judgment, the accuracy of the incoherent events (*M* = 94%, *SEM* = 0.02) was higher than for the coherent events (*M* = 89%, *SEM* = 0.01; *F* (1, 28) = 5.74, *p* < 0.05, $\eta_{p}^{2}$ = 0.17; Supplementary Figure 1A). In contrast, in the picture coherence judgment, the accuracy of the coherent events (*M* = 95%, *SEM* = 0.01) was higher than the incoherent events (*M* = 90%, *SEM* = 0.02; *F* (1, 28) = 4.47, *p* < 0.05, $\eta_{p}^{2}$ = 0.14; Figure 1A). The accuracy of the coherence judgment for the Japanese sentences was not significantly different (coherent events: *M* = 91%, *SEM* = 0.01; incoherent events: *M* = 94%, *SEM* = 0.02; *F* (1,28) = 1.17, *p* = 0.29, $\eta_{p}^{2}$ = 0.04; Supplementary Figure 1A).

A further two-way analysis of variance was conducted to test the effect of coherence judgment of all the conditions on the response time. The significant main effect of the conditions (*F* (2, 56) = 26.95, *p* < 0.001, $\eta_{p}^{2}$ = 0.49) and the interaction effect (*F* (2, 56) = 3.63, *p* < 0.05, $\eta_{p}^{2}$ = 0.11) was found. No significant effect of coherence was found (*F* (1, 28) = 0.03, *p* = 0.85, $\eta_{p}^{2}$ = 0.00). In the picture coherence judgment, the response time of the coherent event (*M* = 0.92 s, *SEM* = 0.03) was faster than that of the incoherent event (*M* = 0.96 s, *SEM* = 0.04; *F* (1, 28) = 9.13, *p* < 0.01, $\eta_{p}^{2}$ = 0.25; Figure 1B). The response times of the coherence judgment of the Chinese sentence (coherent events: *M* = 1.01 s, *SEM* = 0.04; incoherent events: *M* = 0.98 s, *SEM* = 0.04; *F* (1, 28) = 2.22, *p* = 0.15, $\eta_{p}^{2}$ = 0.07) and the Japanese sentence condition (coherent events: *M* = 1.07 s, *SEM* = 0.04; incoherent events: *M* = 1.05 s, *SEM* = 0.04; *F* (1, 28) = 0.37, *p* = 0.55, $\eta_{p}^{2}$ = 0.01) were not significantly different (Supplementary Figure 1B).

Although the accuracy of the coherence judgment of the Japanese sentences showed no significant differences, the accuracy pattern was similar between the Chinese and Japanese sentences, for which the accuracy of incoherent events was higher. On the contrary, in the picture condition, the accuracy pattern was reversed; therefore, the accuracy of the coherent events was higher. This reversed pattern between language and picture was also seen in the response times. Accordingly, it suggests there are differences between the language and the picture judgment, whereas common processing system exists across languages.

Because the conditions had significant effects on response time, the repetition effect on response time was further estimated. All the stimuli were counterbalanced across three scan sessions, e.g., the Chinese sentence stimuli presented in the first scan session determined the order of pictures and Japanese sentences, presented in scan session 2 and 3, respectively (i.e., C-P-J order). Therefore, the Japanese sentence stimuli in the first scan determined the order of Chinese sentences and pictures (i.e., J-C-P order), whereas the order of the picture stimuli was repeated in the Japanese sentence and Chinese sentence order (i.e., P-J-C order). A one-way analysis of variance was used to test for the repetition order differences. The significant effects of the C-P-J order (*F* (2, 56) = 6.61, *p* < 0.01, $\eta_{p}^{2}$ = 0.19) and the J-C-P order (*F* (2, 56) = 21.59, *p* < 0.001, $\eta_{p}^{2}$ = 0.44) were found. In the C-P-J order, the response time of the pictures (*M* = 0.93 s, *SEM* = 0.04) was faster than of Chinese (*M* = 1.07 s, *SEM* = 0.05) and Japanese sentences (*M* = 1.01 s, *SEM* = 0.05) but the response time of Chinese and Japanese sentences were not different (Supplementary Figure 2A). Although, in the J-C-P order, the response time for pictures (*M* = 0.89 s, *SEM* = 0.03) was faster than for Chinese sentences (*M* = 0.96 s, *SEM* = 0.04) and Japanese sentences (*M* = 1.14 s, *SEM* = 0.05; Supplementary Figure 2B). No significant effect of the P-J-C order was found (picture: *M* = 1.00 s, *SEM* = 0.04; Japanese sentences: *M* = 1.03 s, *SEM* = 0.04; Chinese sentences: *M* = 0.96 s, *SEM* = 0.04; *F* (2, 56) = 1.98, *p* = 0.15, $\eta_{p}^{2}$ = 0.07; Supplementary Figure 2C).

Despite the fact that all the stimuli had an equal chance of being presented first, in the picture presented first order (i.e., P-J-C order), there was no significant repetition effect found. This was expected because of the ceiling effect of the repetition effect, as the picture would present sufficient information for food coherence judgment at the beginning. Conversely, in both the C-P-J and J-C-P orders, responses to the stimuli presented later were faster. However, in the C-P-J order, no significant difference was found between the first presented Chinese sentences and the last presented Japanese sentences, which suggests the influence of the second language proficiency.

# Univariate analysis results

Contrasting the coherent condition > incoherent condition using all the data from the three modalities revealed several activated regions, including the left inferior parietal gyrus (BA 40), the right supramarginal gyrus and middle occipital gyrus (*p* < 0.05, FWE-corrected, ke ≥ 5; Supplementary Figure 3A). Alternatively, only a small region of the left calcarine cortex (BA18) was activated for incoherent > coherent condition (*p* < 0.05, FWE-corrected, ke ≥ 5; Supplementary Figure 3B). This result might provide evidence that the left inferior parietal gyrus is specific to coherent event processing.

**Supplementary Figure 1.** Average accuracies **(A)** and response time **(B)** for judging coherence of stimuli of all conditions. Bars represent means with standard errors. C-co represents the Chinese sentence coherent judgment condition, C-inco represents the Chinese sentence incoherent judgment condition, J-co represents the Japanese sentence coherent judgment condition, J-inco represents the Japanese sentence incoherent judgment condition, P-co represents the Picture coherent judgment condition, and P-inco represents the Picture incoherent judgment condition (* *p* < 0.05, ** *p* < 0.01).

**Supplementary Figure 2.** Repetition effect on response time across repetition orders. **(A)** Results for the C-P-J order. **(B)** Results for the J-C-P order. **(C)** Results for the P-J-C order. C represents the Chinese sentence condition, J represents the Japanese sentence condition, and P represents the picture condition (* *p* < 0.05). Bars represent means with standard errors.

**Supplementary Figure 3.** Activated regions for coherent condition > incoherent condition **(A)** and incoherent condition > coherent condition **(B)**. Stable voxel clusters significant at *p* < 0.05, FWE-corrected, extend threshold = 5 voxels.

Supplementary Figure 4. Results of the TFCE correction, showing clusters significant at *p* < 0.05. (A) Results for within-picture classification. (B) Results for across-language classification.
